# Cultural perspectives of older nursing home residents regarding signing their own DNR directives in Eastern Taiwan: a qualitative pilot study

**DOI:** 10.1186/s12904-016-0117-4

**Published:** 2016-05-06

**Authors:** Hsin-Tzu Sophie Lee, Shu-Chen Cheng, Yu-Tzu Dai, Mei Chang, Wen-Yu Hu

**Affiliations:** Department of Nursing, Tzu Chi University of Science and Technology, Hualien City, Taiwan; Department of Nursing, National Taiwan University, Taipei City, Taiwan; Department of Nursing, College of Medicine, National Taiwan University, No. 1, Sec. 1, Jen-Ai Rd., Zhong zheng Dist., Taipei City, 100 Taiwan R.O.C.

**Keywords:** Cultural perspective, Decision-making, End of life (EOL), Nursing home, Do not resuscitate (DNR), Advance directives (ADs)

## Abstract

**Background:**

Chinese tradition and culture developed from Taoism, Confucianism, and Buddhism and have influenced ethnic Chinese for thousands of years, particularly thoughts on death. Many ethnic Chinese, particularly older people, refrain from discussing death-related concerns, making it difficult to obtain advance directives, including do-not-resuscitate (DNR) directives, signed independently by older people. This study explored the attitudes of older nursing home residents in Taiwan toward signing their own DNR directives.

**Methods:**

This study adopted purposive sampling and collected data through in-depth interviews. The data were analysed using qualitative inductive content analysis, and the study location was a nursing home in Eastern Taiwan.

**Results:**

A total of 11participants were recruited from a sample of 12 eligible participants. Most of the older residents in this study refused to make decisions independently regarding DNR directives. Content analysis of the interviews revealed four themes concerning refusing to sign DNR directives independently: not going against nature, accepting the results of cause and effect, viewing the family as a decision-making system, and practising self-effacement. Chinese cultural aspects, including Taoist, Buddhist, and Confucian philosophy, affected the autonomy of the older residents, and they relied on others to make decisions for them.

**Conclusions:**

Professionals must respect this family-oriented decision-making thinking of older residents because it reflects personal choice. Otherwise, healthcare providers may play a mediating role in coordinating and communicating between older residents and their families regarding EOL-care-related concerns, replacing the traditional practice of holding a family meeting.

## Background

Culture not only shapes how people regard health, illness, and death but also plays a central role in decisions concerning end-of-life (EOL) care. Ethnic Chinese refrain from discussing death-related concerns, making it difficult to obtain advance directives (ADs), including do-not-resuscitate (DNR) directives, signed independently by older people [1]. Cultural obligations and particular social and historical experiences govern death-related concerns. Chinese tradition and culture developed from Taoism, Confucianism, and Buddhism. These three main philosophies have influenced the thinking and living of ethnic Chinese for thousands of years [1, 2]. In Taiwan, Taoist, Confucian, and Buddhist philosophies have been integrated into curricula from elementary school to university. Therefore, these three philosophies are not regarded as religion but rather as a set of attitudes for daily living, attitudes that provide an overarching framework for values and beliefs in Taiwan, particularly for death-related thinking [2].

Considered a philosophy, Taoism emphasises people’s connection to natural life forces [3]. Taoism proposes that people seek harmony with nature rather than attempt to change it [2]. Its concepts advocate that humans recognise the patterns of nature, enjoy harmony and inner peace of mind, and avoid disrupting the flow of natural cycles. The life philosophy of Taoism primarily entails following the pattern of nature and doing nothing the sake of one’s life [4]. Although it does not discuss death, Confucianism provides the basis for the Chinese moral code and behavioural ethics, such as filial piety and familism. A disciple asked Confucius regarding thinking about death, and Confucius answered, ‘While you do not know life, how can you know about death?’ [5]. Therefore, according to Confucius, ethnic Chinese must sacrifice to uphold their morals and make their families proud of them rather than worry about dying. Ethnic Chinese in general and patients and older people in particular regard thinking or talking about death or dying as taboo [3]. For ethnic Chinese, discussing death-related concerns in front of or with older people is considered a curse that will bestow bad luck on them [3, 4]. Buddhism has greatly influenced Chinese culture and is the basis for the spiritual rituals practiced by ethnic Chinese. Samsara and karma are the two main beliefs of Buddhism. Samsara means the ‘wheel of life’, referring to the cyclical stages of existence as a person progresses through reincarnation (birth-death-rebirth) [2, 3]. Karma refers to the merit accumulated by doing merciful acts, which leads to an improved life in a person’s next reincarnation [1]. The concepts of samsara and karma in Buddhism are not only related to the individual but are also adapted to include ancestors and families [3]. Therefore, ethnic Chinese attribute their fate to samsara and karma.

Influenced by Confucian, Taoist, and Buddhist philosophies of life, ethnic Chinese believe that death and life are predetermined and that an individual must not plan for or discuss EOL-care-related concerns [1]. Thus, ethnic Chinese tend to comply with the natural course of life and the will of their families, respect their fate and karma, and avoid making independent decisions [4]. Compared with older Europeans and Americans, older Taiwanese residents in long-term care facilities have less control over decisions concerning their own health and feel strongly that they must consult their families before making any EOL-care-related decisions [1]. Studies from Taiwan have reported that all decisions for older people, from living in long-term care facilities to receiving any type of medical treatment, were made by their families. Most older people were sent directly to live in nursing homes without being informed by their families, and approximately 91–98 % of decisions pertaining to EOL care were made by families [6–8]. Lo proved that most EOL-care-related decisions, such as receiving life-sustaining therapy or signing DNR directives, were made by families (98.5 %, *n* = 198/201). Most families (83.6 %, *n* = 168/201) chose life-sustaining therapy to prolong the life span of their parents because of filial piety [7]. In other words, most children select life-sustaining treatments when their older parents are nursing home residents and leave EOL decision making to them; such treatments tend to be more aggressive and cost the Taiwan government’s public health insurance program a substantial amount to assist these adult children in upholding the tradition of filial piety, an undesirable outcome for the government [8, 9]. In addition, Chen et al. showed that, although families play a primary role in decision making regarding life-sustaining treatments, one-half of the families thought that this decision should be made by the patients themselves and desired to know what their parents thought of the decision making but preferred that the healthcare provider ask them [10].

However, previous studies have point to a gap in the research, a phenomenon where older residents in long-term care facilities were highly willing but did not sign their own DNR directives [4, 7–9, 11]. In addition, studies have confirmed that education programs on advance care planning (ACP) increased ADs knowledge among older residents but that the rate of ADs completion remained low [12, 13]. Therefore, previous studies have indicated that attitude-related barriers to completing ADs must be evaluated in detail before implementing ACP [13–15]. Nevertheless, only two studies thus far have explored the perspectives of older residents in long-term care facilities in Taiwan regarding signing their own DNR directives; their results showed that more than 90 % of older nursing home residents rejected to sign their own DNR directives because of either familism or concern about fearing death [7, 9]. The aim of this study was to explore the perspectives of older nursing home residents in Taiwan regarding signing their own DNR directives.

## Methods

### Ethical review

This study was approved by the Research Ethics Committee of Hualien Tzu Chi Hospital, Buddhist Tzu Chi Medical Foundation (reference number: IRB100-19) on 21 May 2012.

### Setting

Five nursing homes are in the Hualien City area. They are typically small nursing homes with mostly older residents who either have dementia-related problems or are in a vegetative state. The research setting selected for this study was the only nursing home near Hualien City where more than 10 residents had Mini-Mental State Examination (MMSE) scores ≥ 24. The nursing home was located in Eastern Taiwan, and interviews were conducted over a span of 6 months. Because the nursing home had no ethics committee, ethics approval was obtained from the ethics committee of the largest hospital in Eastern Taiwan.

### Sampling

This research setting was a small nursing home with approximately 40 residents. Consecutive sampling was used for participant selection. Potentially eligible older nursing home residents were selected using the following inclusion criteria: (1) residence in the nursing home for >1 month, (2) an age ≥ 65 years and ability to speak Mandarin Chinese or Taiwanese, (3) an MMSE score ≥ 24, and (4) the willingness and ability to share feelings with the researchers about signing a DNR directive. After confirming the eligibility criteria with each older nursing home resident, the first author (Lee) would ask the resident whether he or she was willing to participate in this study. The participants were added to the sample until data saturation was reached.

### Study procedure

Before conducting this study, the first author worked weekends with a staff nurse at the nursing home for 1 year to provide care to the older residents and have conversations with them. This work experience enabled the first author to identify potentially new knowledge embedded in the care context and to develop an interview guide. After gaining a general understanding of the concerns of the older nursing home residents about signing their own DNR directives, the first author (Lee) developed an interview guide and discussed it with the fifth author (Hu). Consensus regarding the interview questions was achieved, and the interview guide was finalised (Table 1).

**Table 1:** Interview guide

1.	Why do you live here? How do you feel about living here?
2.	How many chronic diseases do you have? Can you tell me more about your present health condition?
3.	What is your opinion about the treatment you are presently receiving for these diseases?
4.	Are you satisfied with the treatment or management provided by your doctors? Why?
5.	What is your opinion about your past, present, and future life?
6.	What is your opinion about death?
7	What is entailed in a good death?
8.	What is your opinion about hospice care?
9.	If your condition becomes severe, what type of care or treatment would you choose?
10.	If your condition becomes severe, will you make decisions about end-of-life treatment by yourself? Why?
11.	What are your opinions and your family members’ opinions about treatment related to end-of-life care? Have you discussed these issues?
12.	In what type of situation would you like to discuss issues related to end-of-life treatment or care with your family?
13.	If you told your family members that you wanted to sign your consent to your own do-not-resuscitate directives, what would be their reaction? Would they respect your decision?

Formal interviews were conducted in the nursing home, with all interviews being conducted by the first author (Lee) in Mandarin or Taiwanese. Each participant received an in-depth, face-to-face interview lasting 30–40 min with open-ended standard questions. The interviews were recorded on tape and transcribed verbatim shortly afterwards. Conversation notes were recorded immediately, and field notes on paper were transcribed via a personal computer on the same day. The researcher collected data through observation during the nursing home visits. Twice a month, the interviewer (Lee) and other researchers met to discuss the recent interview results. Field notes and interview transcripts were the focus of these discussions, and the discussions provided the basis for code analysis.

### Data analysis

This study adopted purposive sampling, conducted in-depth interviews, and performed qualitative, inductive content analysis. Study data were preserved in field notes and interview transcripts, which were jointly reviewed by all members of the study team twice monthly. The qualitative, inductive content analysis method used was based on guidelines by Holsti [16] and by Graneheim and Lundman [17]. The transcribed content of the interviews and field notes from the visits with each participant was analysed as follows:(1) The interview transcripts and field notes for each participant were written and read numerous times by the first author to understand the content. (2) After the statements and units of responses were identified, constellations of words, phrases, and sentences relating to the same central meaning were divided independently into meaningful units by the first and second authors (Lee and Cheng) and were discussed with and revised by the fifth author (Hu). (3) The meaningful units were condensed into ‘condensed meaning units’, which are descriptions that are similar to the text and based on an interpretation of the underlying meaning. These condensed meaning units were compared and abstracted into nine subthemes according to their similarities and differences and were agreed on by all authors during the meetings. These subthemes were subsequently presented to the participants and revised according to their opinions. (4) Finally, a discussion with all authors resulted in agreement on an emerging set of meaning units, condensed meaning units, subthemes, and themes.

## Results

A total of 11participants were recruited from a sample of 12 eligible participants whose MMSE scores were ≥24. The primary reason for excluding one of the 12 eligible participants was that the participant was being relocated to another nursing home after being discharged from the hospital. The age of the participants in the sample ranged from 65 to 92 years (mean, 79y), and most participants were women (*n* = 7, 64 %). Of the participants, one had a college degree, seven had completed elementary school, and three were illiterate. The average number of chronic conditions among the participants was 3.5; the five most common chronic diseases were hypertension (73 %), arthritis (64 %), diabetes mellitus (45 %), heart disease (45 %), and stroke (45 %). The religious affiliations of the residents were Buddhist (*n* = 7), Taoist (*n* = 3), and Catholic (*n* = 1). On average, the participants had lived in the nursing home for 5 years (Table 2). Because the nursing home is a private institution, residents’ families pay NT$40–60,000 per month, without governmental support, to house an older resident there, which is quite a high cost for a Taiwan family to afford. Consequently, the residents had favourable economic statuses.

**Table 2:** Participant characteristics (*n* = 11)

Characteristics	N (%)
Gender	
Male	4 (36)
Female	7 (64)
Age	
65–69 years	3 (27)
70–74 years	1 (9)
75–79 years	1 (9)
80–84 years	2 (18)
85–89 years	2 (18)
> 90 years	2 (18)
Education status	
College	1 (9)
Elementary school	7 (64)
Illiterate	3 (27)
Religious affiliations	
Buddhist	7 (64)
Taoist	3 (27)
Catholic	1 (9)
MMSE scores	
28–30	2 (18)
27–24	9 (82)
< 24	0 (0)
Resident relationship with primary caregivers	
Children (son/daughter)	9 (82)
Siblings (sister/brother)	2 (18)
Chronic diseases	
Hypertension	8 (73)
Arthritis	7 (64)
Diabetes mellitus	5 (45)
Heart disease	5 (45)
Stroke	5 (45)
Kidney disease	2 (18)
Cancer	2 (18)
Anxiety	2 (18)
GI disorder	1 (9)
Asthma	1 (9)
Duration of living in nursing home	
< 1 year	3 (27)
1–5 years	6 (55)
> 5 years	2 (18)
Average monthly income of participant families	
> NT$20,000/per month	8 (73)
NT$15–20,000/per month	3 (27)
< NT$15,000/per month	0 (0)

The results of the qualitative, inductive content analysis [16, 17] showed that one resident wanted to sign his own DNR directive but that the rest of the participants (*n* = 10, 91 %) refused to make independent decisions about EOL care. Among these 10 participants, eight of them firmly refused to sign their own DNR directives, and the remaining two expressed that they wanted to sign but needed the permission of their children. Content analysis of the interviews revealed four themes regarding the participants’ refusal to sign their own DNR directives: not going against nature, accepting the results of cause and effect, viewing the family as a decision-making system, and practising self-effacement (Table 3).

**Table 3:** Themes, subthemes, and meaning units regarding older residents regarding refusing to sign their own DNR directives

Content of themes	Content of subthemes	Condensed meaning units
Not going against nature	1. Over-thinking	(1) Unnecessary things
		(2) Useless things
	2. Natural life	(1) Taking it as it comes
		(2) Following the mandate of nature
Accepting the results of cause and effect	1. ‘That’s karma’	(1) Do good and receive good
		(2) Fate would have it so
	2. ‘That’s samsara’	(1) The time has not arrived to discuss
		(2) Fate is fulfilled (by what you have done in your present and past lives)
Viewing the family as a decision-making system	1. Thoughts of filial piety	(1) Fulfilling the value of filial piety
		(2) The responsibility of children
	2. Familism	(1) A sense of belonging to family
		(2) Family-centred decision making
	3. Death and EOL care as a taboo	(1) A misfortunate word becomes reality
		(2) Causing problems for family
Practising self-effacement	1. Everyone does his or her own part	(1) Making decisions for patients is doctors’ responsibility
		(2) Shifting the responsibility to others
	2. None of my business	(1) Having no ability to make one’s own decisions
		(2) Everyone knows what to do

### Not going against nature

Most participants refused to answer questions about the type of EOL care that they wanted or their willingness to sign their own DNR directives and regarded those concerns as over-thinking. Furthermore, they expressed that death is a natural event and that a human must seek harmony with nature rather than try to change it.

#### Over-thinking

Most elder nursing home residents regarded independently signing DNR directives as unnecessary. They expressed a strong urge not to think about death or dying because they believed that living happily in the long-term care facility had priority over thinking about death.

*“It is unnecessary and ridiculous for me to think about whether to receive CPR or not…or…what kind of care I want to have at the end of life right now…the only thing I want to do right now is to live here happily and smoothly”* (Informant 10).

*“Death is death, although you think about how to die every second…death will come…it can’t be changed, and you don’t know when you will die…so…it is useless to think about it…just forget it*” (Informant 2).

*“It is unnecessary to make a decision about what kind of care I want to have during the end of life because death is death…nothing else”* (Informant 6).

#### Natural life

The participants believed that, when their time was due, they would face the situation bravely and follow the course mandated by nature, as a tree does that has accepted its situation without questioning.

*“I was a sailor, and I have seen too much…when your time is coming…you will die…and how you will die has been decided by providence already…whether you will be killed by a pirate or a shark…can’t be predicted…it is natural and can’t be changed”* (Informant 8).

*“I don’t want to think or talk too much about EOL care…just let it happen naturally…everything must follow the mandate of nature… just as that tree outside the window accepts its situation from nature without questioning”* (Informant 3).

*“You cannot go against nature…everything must follow the mandate of nature…why do you need to plan about end-of-life care?”* (Informant 5).

*“I don’t want to have CPR at the terminal stage to prolong my life; that is not natural…when the time comes, just let me go…to die is natural…it’s a natural part of life, isn’t it?”* (Informant 7).

### Accepting the results of cause and effect

Most participants in this study refused to sign their own DNR directives or discuss EOL care because they were influenced by certain Buddhist ideologies, such as karma and samsara.

#### ‘That’s karma’

Most participants responded to questions regarding the type of EOL care that they would receive in terms of fate (Chinese: *min*命). If a person has performed good deeds in his or her past or present life, that person will die a peaceful death; however, according to karma, that person will not die peacefully if he or she has not performed good deeds. The residents strongly believed in the concepts of ‘doing good and receiving good’ and ‘fate making it so’.

*“An old saying goes, ‘a bad penny always comes back.’ I am a good person…I treat my family, friends, and everyone very kindly…I am also a devout Buddhist and…therefore… I never worry about death”* (Informant 4).

*“God knows best for me, and he will bless me with everything…and I will also accept any situation from him happily…if he thinks I need to have CPR at the end of my life before going to see him…it is my destiny to accept this challenge from him”* (Informant 6).

#### ‘That’s samsara’

The participants regarded signing their own DNR directives to be a foreign concept because they believed that, according to samsara, the time and manner of their death had been predetermined by their actions in their present and past lives. Some participants stated that thinking about how to die is unnecessary because the time has not arrived to discuss these concerns. Other participants believed that how a person dies has been predetermined and that fate is fulfilled according to providence, which depends on a person’s actions in present and past lives.

*“Because…I am healthy presently and too young to think about issues related to death or EOL care…would you please not talk about these issues until I am more than 70 years old?”* (Informant 10).

*“Let me tell you…when you will die and how to die…these things have been decided already by what you have done in present and past lives…it is really complex…we may never be able to understand the language of providence”* (Informant 2).

### Viewing the family as a decision-making system

Most participants declined to sign their own DNR directives because of filial piety, a sense of belonging to family and a concern about causing problems for family.

#### Thoughts of filial piety

Most participants regarded making EOL-care-related decisions for parents, such as signing a DNR directive, to be a filial behaviour of children and strongly believed that their children would know the most appropriate option for them. Some participants mentioned that making EOL-care-related decisions for parents was a major responsibility of children.

*“I know that my son cares about me, and I trust him…if he wants me to live longer, let me have CPR; if he doesn’t want me to suffer too much pain and wants me to pass away, I would also agree with him…he knows which decision is best for me…that is the value of filial piety to me”* (Informant 3).

*“When I was young, I also made every decision for my parents…right now, I am an old man…my children have to take this responsibility from me…this is why I need to have children, right? I don’t want to do their job”* (Informant 4).

#### Familism

Childless participants wanted to leave the right of signing their DNR directives to a brother or sister; this sense of belonging to family was extremely valuable to them.

*“When my parents got very sick, we all made decisions for them together…although I didn’t get married, I still have two brothers…I hope I can leave these decisions to them to make together…it’s our culture”* (Informant 8).

*“I am afraid that if I sign these documents without their permission, it will make them feel terrible and angry with me…I need to consider their feelings…it is not right for me to make this important decision by myself without their permission”* (Informant 11).

#### Death and EOL care as a taboo

Not wanting to cause problems, no participant wanted to discuss death or EOL care with family. Most of the participants and their children believed that discussing EOL-care-related concerns would bring them bad luck. Therefore, children or relatives never mentioned death or dying in front of the participants. Although some residents tried talking to their children about matters related to their eventual passing, their children would immediately stop their parents, telling them that the subject made them too sad or scared them and that they did not want to continue the conversation.

*“When I was a child, my parents told me…not to talk about death or dying with older people…it will seem like you hope that they die soon…it will bring bad luck”* (Informant 4).

*“Death is just death…why should I worry right now? If I talk to my family about EOL care…it will seem that I am threatening my family and making them feel sorrow…I don’t want to do this”* (Informant 2).

### Practising self-effacement

Most participants in this study refused to sign their own DNR directives because they strongly believed that they were incapable of making this decision for themselves and required someone else to do it.

#### Everyone does his or her part

Most participants expressed that they did not worry about EOL care because everyone performed their jobs effectively. The participants stated that the role of doctors was to make decisions for their patients; thus, when the participants experienced a life-threatening event, doctors would make the most beneficial decision for them. In addition, when making decisions concerning EOL care or signing a DNR directive, most participants entrusted others to make the decisions on their behalf and believed that these people made appropriate decisions.

*“Doctors are professionals…if doctors think I can’t be saved…just let me die soon…if doctors think I will have the chance to live longer…let me have CPR…I have told my children that they need to trust doctors’ ability and follow their orders without question”* (Informant 1).

*“When the time comes…I will be sent to the hospital in an ambulance…doctors will decide whether or not to give me CPR…and my children will sign the papers…the only thing I need to do is lie there unconscious… so, what else do I need to do?”* (Informant 8).

#### None of my business

The participants regarded signing their DNR directives or making decisions related to EOL care to be the responsibility of others. Most participants stated that they lacked the ability to make decisions; they believed that everyone, including their children, doctors, and even the nursing home staff, was smarter than they were and that matters related to death and dying were too critical and complex for them to make decisions independently.

*“I have no idea about things related to EOL care or signing DNR papers…they are too complex for me to make decisions by myself without their [my children’s] permission…so, please ask my children…they are smarter than I am, and they can make any decision for me by themselves”* (Informant 7).

*“It is not necessary for me to sign the DNR directives or make decisions about EOL care myself…when the time comes, my children, the doctors, the staff in this nursing home, and you [first author] will know what is best for me…the only thing I need to do is accept it…”* (Informant 2).

## Discussion and conclusion

Several decisions must be made when considering EOL care options, and the decision maker is largely influenced by cultural values [1, 18]. Influenced by Buddhism, Taoism, and Confucianism, ethnic Chinese traditionally regard discussing death or related topics with older people as taboo. Therefore, older people in Taiwan tend not to make their own EOL care decisions or sign any type of ADs, including DNR directives [4, 9]. In this study, 91 % of the participants perceived signing ADs as irrelevant because they intended not to go against nature, accepted the results of cause and effect, viewed the family as a decision-making system, or practised self-effacement. These behaviours are clear manifestations of the traditional beliefs and cultural customs, developed from Taoist, Buddhist, and Confucian philosophies, that are upheld by ethnic Chinese.

Consistent with previous studies [4, 9], two-thirds of the participants in this study expressed strong feelings against signing their own DNR directives and did not intend to make any plans regarding EOL care; they believed that everything must follow the course mandated by nature and that people should not disrupt this natural flow, which could be disrupted by behaviours such assigning their own DNR directives in advance. Because of Taoist influences, ethnic Chinese believe that death and life are predetermined and hence do not require planning or discussion. A comparison of studies on EOL attitudes in Taiwan and New Zealand indicated that approximately half of the ethnic Chinese participants agreed that the time of a person’s death is predetermined and that it is necessary to live in harmony with nature rather than attempt to change it [4]. However, the remaining one-third of the participants (*n* = 3) in this study thought that having cardiopulmonary resuscitation (CPR) at the terminal stage was against the mandate of nature; they preferred to die naturally. However, two of them still refused to sign their DNR directives in advance without family permission; they also wanted the healthcare provider to be the bridge between them and their children. Previous studies have not mentioned results indicating that participants viewed life-prolonging CPR at the terminal stage to be behaviour that went against nature and that participants wanted healthcare providers to communicate their EOL thoughts to their families. For older ethnic Chinese nursing home residents, we suggest that professionals should first interview residents to determine their thoughts regarding EOL care and then inform their families of those thoughts.

On the basis of Buddhist philosophies, ethnic Chinese believe that everything has been predetermined because of karma (cause and effect). The results of cause and effect can be attributed to a person’s previous life, with the results experienced in the current life affecting the next life [2, 3]. Most participants in this study refused to sign their own DNR directives or discuss EOL care because they were influenced by certain Buddhist ideologies. Their thoughts on attributing actions to effects and believing that every action is predetermined reflect concepts embodied in Buddhist thinking. This finding supports those of Hsu et al., who observed that older Taiwanese people respected their fate and attributed their future to karma and samsara [3].

In addition, previous studies have indicated that ethnic Chinese have family-centred thinking and tend to regard the family as a decision-making system [1, 3, 4, 7–9]. Similarly, the findings of this study revealed that most participants declined to sign their own DNR directives because of filial piety, a sense of belonging to family and a concern about causing problems for family. These thoughts reflect concepts embodied in Confucian teaching. Confucianism emphasises the virtues of filial piety, family values, and familial as well as social responsibilities [3, 19]. Ethnic Chinese are educated that the family should be considered before the individual; ethnic-Chinese families identify closely with each other [1]. However, previous studies have not attributed ethnic Chinese avoiding discussions on EOL care to Confucian teachings that emphasise sacrificing someone’s life to achieve personal moral development. In this study, most participants regarded talking or thinking about EOL care as taboo. According to Confucian teaching, humans must sacrifice themselves to achieve personal moral development and make their country and family proud [2, 3]. Therefore, followers of Confucianism are discouraged from discussing topics such as disorders or death that might weaken their willingness to sacrifice. Ethnic Chinese strongly believe that discussions about death bestow them with bad luck. A common general strategy for coping with fear of dying is avoiding the topic [3]. Consequently, the participants considered independently signing their own DNR directives to be an extraordinary concept. Most participants in the present study refused to sign their own DNR directives because they thought that planning for death and dying was a foreign concept, did not intend to trouble their families, and wanted to maintain family harmony.

The concept of self-effacement, a result of mixing the philosophies of Buddhism, Taoism, and Confucianism, is unique among ethnic Chinese. They are taught by their parents and teachers that they should not live for themselves but for family, society, and country [1–3, 19]. In Chinese philosophies, the concept of ego does not seem to exist, and ethnic Chinese tend to pass decision-making authority to family members, healthcare professionals, and facility staff. This point has not been mentioned in previous studies. The tendency to pass decision-making authority to family members has been attributed by most researchers to a pattern of family-centred decision making [4, 19]. However, this description does not explain the findings of the present study. Most participants refused to sign their own DNR directives because they strongly believed that they were incapable of making this decision for themselves and required someone else to do it. Certain older residents in this study even asked the first author to make such decisions for them.

This study showed that these three main philosophies induced a phenomenon where most of the older nursing home residents refused to make EOL decisions independently and where some of them expressed the need of obtaining permission from their families to sign their own DNR directives. Similarly, a study by Fan and the author of this article indicated that, ‘for East Asian people, these issues (about death or dying) are too important to be left only to oneself, even if one is competent’ [20]. Therefore, we suggest that professionals respect this pattern of family-determined or professional-oriented decision-making from older residents because it is a decision they have made that reflects personal choice. However, under the influence of Western culture and changing socioeconomic circumstances, the thoughts of families in Taiwan about making EOL care decisions for their parents or relatives have changed imperceptibly. In Chen et al., 56 % of the families thought that the patient should make decisions about life-sustaining treatment and expressed that they would follow the patient’s decisions [10]. However, because of the influence of culture, the families and older residents in this study never communicated with each other about EOL care decisions; both of them wanted the healthcare providers to take the responsibility of communicating about such issues but to talk to each of them separately. On the basis of these study results, we suggest that professionals interview older residents and then inform their families of their EOL thoughts. Concurrently, professionals can also provide families with suggestions about ELO care according to medical ethical principles such as the principles of beneficence and nonmaleficence to assist them in making EOL decisions for the older residents.

### Implications for clinical practice

The findings of this study demonstrate how culture can influence people, particularly older people. Concerns regarding death and dying are culturally constructed through specific social and historical experiences. The role of family for ethnic Chinese is too crucial to be replaced or ignored. Professionals must respect this family-oriented decision-making thinking of older residents because it reflects personal choice. Moreover, healthcare providers may play a mediating role regarding EOL-care-related concerns by coordinating and communicating between older residents and their families, replacing the traditional practice of holding a family meeting. Thus, healthcare professionals not only assist families in having death-related discussions with older residents but also prevent the older residents from experiencing negative feelings such as shock and depression that could occur were family members to have a direct discussion with them regarding their EOL care decisions.

### Study limitations

This study has several limitations. The first regards the sampling setting. The nursing home was small; however, the quality of the services was high, and most residents had favourable economic statuses. In addition, the older residents had amicable relationships with their families. Consequently, it was difficult to determine whether different economic backgrounds or the older residents’ relationships with their families affected their attitudes toward signing their own DNR directives. The second limitation relates to courtesy bias. The location of this nursing home is neither near Tzu Chi University of Science and Technology nor near Tzu Chi Hospital; therefore, this nursing home has no relationship with any Tzu Chi organisation. However, the name ‘Tzu Chi’ is extremely famous in Taiwan because of its association with a Buddhist philanthropic organisation; and, when first interacting at the nursing home, the first author did introduce herself as a teacher from Tzu Chi University of Science and Technology. Hence, the interviewer’s background might have influenced the conventional views expressed by practitioners and caused courtesy bias.

### Ethics and consent to participate

This study was approved by the Research Ethics Committee of Hualien Tzu Chi Hospital, Buddhist Tzu Chi Medical Foundation (reference number: IRB100-19). The participants provided written informed consent; a consent-to-participate statement was included under the “Ethics, consent and permissions” heading in the participation document and a statement under the “Consent to publish” heading granted the author consent to publish individual participant data and information obtained from participant data.

### Availability of data and materials

The dataset supporting the conclusions of this article is included within the article. However, the raw dataset cannot be shared because of a rule of the Institutional Review Board of the Research Ethics Committee, Buddhist Tzu Chi Medical Foundation.

## Abbreviations

ACP: advance care planning
ADs: advance directives
CPR: cardiopulmonary resuscitation
DNR: do not resuscitate
EOL: end of life
